# Comparative effect of transforaminal injection of Magnesium sulphate versus Ozone on oxidative stress biomarkers in lumbar disc related radicular pain

**DOI:** 10.1186/s12871-022-01789-0

**Published:** 2022-08-08

**Authors:** Wael Fathy, Mona Hussein, Raghda E. Ibrahim, Manar M. Abdel-Aziz, Shaden Adel, Shaimaa H. Soliman, Hatem Elmoutaz, Mohamed Abdelkader

**Affiliations:** 1 Department of Anaesthesia, Surgical ICU and Pain Management, Beni-Suef University, Beni-Suef, Egypt; 2 Department of Neurology, Beni-Suef University, Beni-Suef, Egypt; 3 Department of Clinical and Chemical pathology, Beni-Suef University, Beni-Suef, Egypt; 4 Department of Psychiatry, Cairo University, Cairo, Egypt; 5 Department of Neurology, Cairo University, Cairo, Egypt

**Keywords:** Lumbar disc prolapse, Mg sulphate, Ozone, GSH, SOD

## Abstract

**Background:**

We aimed to investigate the effect of transforaminal injection of Magnesium sulphate versus Ozone on pain intensity, functional disability and the oxidative stress biomarkers; superoxide dismutase (SOD) and Glutathione (GSH) in patients with lumbar disc prolapse.

**Methods:**

This randomized controlled trial was conducted on 135 patients having symptomatic lumbar disc prolapse, received either transforaminal injection of Magnesium sulphate with steroids, Ozone with steroids, or steroids alone. Assessment of pain severity and functional disability were done before intervention, 2 weeks, 1, 3, and 6 months after intervention. Serum SOD and GSH were measured for all included patients before and 2 weeks after intervention.

**Results:**

There was a statistically significant improvement in pain intensity and functional disability 2 weeks after intervention in the three groups, but at 1-month and 3-months after intervention, the significant improvement was in Mg sulphate and Ozone groups only. At 6-months follow up, Mg sulphate group only showed a significant improvement. There was a statistically significant increase in SOD and GSH serum levels, 2-weeks after intervention in both Magnesium sulphate (*P*-value = 0.002, 0.005 respectively) and ozone groups (*P*-value < 0.001, < 0.001), but there was no statistically significant change in SOD and GSH serum levels in control group.

**Conclusion:**

Transforaminal injection of Mg sulphate in patients with lumbar disc prolapse causes significant long-term improvement (up to 6 months) in pain intensity and functional disability. The serum levels of SOD and GSH were significantly increased at 2 weeks following both transforaminal injection of Mg sulphate and ozone.

## Introduction

Lumbar disc prolapse is one of the most common causes of low back pain and/or radicular pain. The pathogenesis of lumbar disc related radicular pain is likely related to mechanical and/or inflammatory factors. The natural history of lumbar disc prolapse is mostly favorable, with about 80–90% of patients showing improvement within 1- 3 months [1, 2]. Management is usually directed medically to analgesic, anti-inflammatory and neurotonic drugs [3]. Other minimally invasive approaches were evolved such as transforaminal injection of steroid, magnesium sulphate and ozone [4].

Magnesium sulfate has drawn much attention in the field of pain management, resulting in numerous publications of review articles, clinical trials, and meta-analyses [5]. Intravenous administration of magnesium sulphate was reported to be effective in the management of neuropathic pain [6]. Much concern was directed towards studying the therapeutic effectiveness of neuraxial administration of magnesium sulphate. Intrathecally-administered magnesium sulphate was found to be free of neurotoxicity. The safety of epidurally-administered magnesium sulphate was also reported [7, 8].

Ozone (O_3_) therapy has been emerged in the treatment of neuropathic pain, inflammatory, degenerative and herniated disc conditions [9]. It activates different antioxidant response elements including glutathione (GSH), superoxide dismutase (SOD), and catalase (CAT). These enzymes are considered free radical scavenger [10].

Ozone therapy produces its analgesic and anti-inflammatory effects through decreasing the stimulation of sensory nerve endings by inflammatory mediators and inhibition of peripheral sensitization. It also has an antiseptic and immunomodulatory function [11].

Strong evidence suggested an essential role for oxidative stress in the pathophysiology of disc degeneration and herniation. In the disc tissues, oxidative stress can initiate matrix destruction and cell apoptosis, leading to a disc degeneration [12]. So, using antioxidants in patients with lumbar disc prolapse may be a promising therapeutic approach that may prevent or retard the progression of disc degeneration [13]. To our knowledge, the beneficial effect of transforaminal injection of antioxidants in patients with lumbar disc prolapse, was not previously investigated.

The aim of this work was to investigate the therapeutic effect of transforaminal injection of Magnesium sulphate versus Ozone on pain intensity, functional disability, and the oxidative stress biomarkers; SOD and GSH in patients with symptomatic lumbar disc prolapse.

## Methods

### Study design and participants

This prospective randomized controlled trial was carried out on 135 patients diagnosed as having symptomatic lumbar disc prolapse. Patients were randomly assigned into one of three groups; the first group received transforaminal injection of Magnesium sulphate with steroids (45 participants) (Magnesium sulphate group), the second group received transforaminal injection of Ozone with steroids (45 participants) (Ozone group), and the third group received transforaminal injection of steroids alone (45 participants) (Control group). Randomization was performed using the opaque closed envelope technique where the clinician picked up a sealed envelope containing a sheet of paper with the name of the group to which the patient was randomly selected. Whichever group was written on the sheet, the patient was scheduled to it. The patients were recruited from the neurology and pain clinics of Beni-Suef University Hospital, in the period from November 2020 to November 2021. The study was registered in ClinicalTrials.gov on 24/9/2020 and this is the identification number NCT04562493.

### Eligibility criteria

The study included patients with clinical evidence of disc bulge in the form of lumbar disc related radicular pain (radiating pain in the lower limb that follows a dermatomal pattern) of >3 months duration, not responding to conservative treatment (medical treatment and physiotherapy) and interfering with daily activities. The selected patients must have radiological evidence of posterolateral lumbar disc bulge by MRI lumbosacral.

**Fig. 1:**
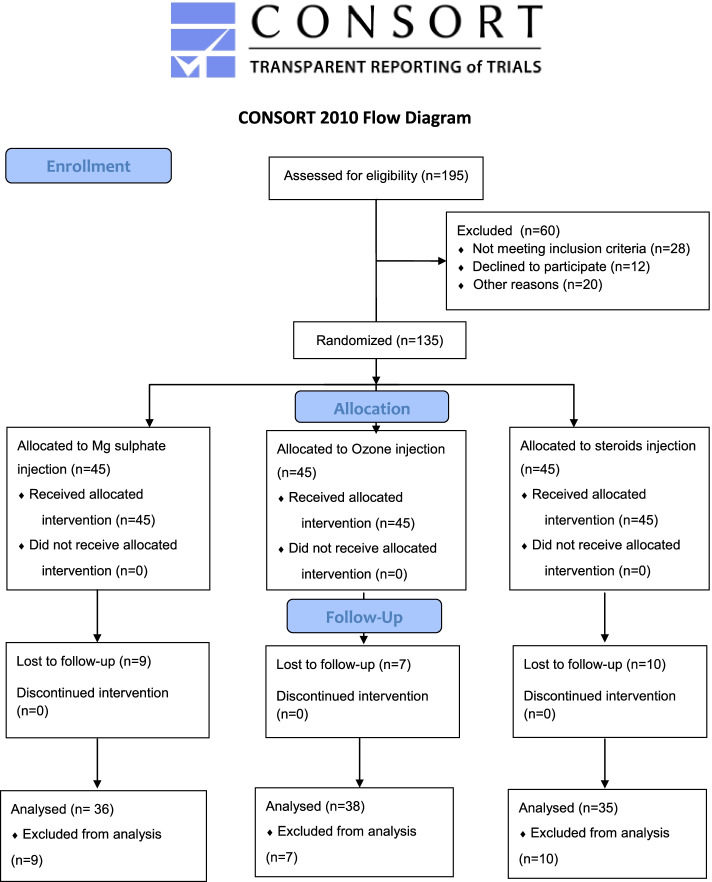
CONSORT flow diagram

The following patients were excluded from our study: patients with a history of spinal trauma, spinal surgery or spinal deformities, patients with radiological evidence of any inflammatory or neoplastic lesion affecting the vertebral column, spinal cord or the surrounding soft tissue, patients with clinical or radiological evidence of hip osteoarthritis, lumbar zygophysial joint arthritis [localized pain over the lumbar vertebrae that worsens with standing or bending backward and is typically relieved by bending forward], or sacroiliitis [low back or buttock pain with typical increase in severity at night and associated stiffness upon awakening, with some amelioration after exercise, and one of the following provocative tests should be positive: pelvis rock test, FABERE (Flexion, ABduction, External Rotation, Extension), and Gaenslen maneuvers]. We also excluded patients with severe lumbar disc prolapse causing lower limb weakness or sphincteric troubles, and patients with contraindications to interventions (sepsis, coagulopathy, or allergy from the used drugs). Pregnant patients were also excluded from our study. We demonstrated in a flow diagram for the recruited patients that 195 patients were assessed for eligibility. Sixty patients were excluded (28 patients didn’t meet inclusion criteria, 12 patients declined to participate, and 20 patients were excluded due to other reasons). Forty-five patients in each group received allocated intervention. Nine patients in Mg sulphate group, seven patients in Ozone group, and 10 patients in control group lost to follow-up (Fig. 1).

### Data collection

History was taken from the selected patients regarding the demographics and the duration of lumbar disc related radicular pain. The imaging findings regarding the number of prolapsed discs and the degree of the most prolapsed disc were also obtained.

### Assessment of pain intensity and functional disability

Assessment of the pain intensity and functional disability was done before and 2 week, 1, 3, and 6 months after the interventional procedure by a neurologist who was blinded to the type of intervention. Assessment was done using Numeric Rating Scale (NRS), Oswestry Disability Index (ODI), and Functional rating index (FRI).

NRS is a single 11-point numeric scale for assessment of intensity of pain. It ranged from 0 to 10, where 0 indicates no pain and 10 indicates the worst pain [14].

ODI is a questionnaire used to quantify the disability from low back pain. It includes the following items: pain intensity, the level of disability of personal care, lifting, walking, sitting, standing, sleeping, traveling, sexual and social life. Each question is scored on a scale of 0–5 with 0 representing no limitation, and 5 representing maximal limitation. The scores for all questions answered are summed, then multiplied by two to obtain the index (range 0 to 100) [15].

FRI is a self-reported scale containing 10 items. Eight items focus on daily activities affected by back pain and 2 items focus on frequency and intensity of pain. Each item has a 5-point scale (0 = no pain, and 4 = worst possible pain). The FRI scores ranged between 0% (no disability) and 100% (severe disability) [16].

### Assessment of patients’ satisfaction

Assessment of patient’s satisfaction about the intervention was done 6 months after the interventional procedure by a psychiatrist who was blinded to the patient’s condition and the type of intervention. It was done by using the Short Assessment of Patient Satisfaction (SAPS) scale. SAPS consists of the following seven items: satisfaction with treatment, explanation of the treatment outcome, medical care, respect by the physician, participation in medical decision making, time with the physician, and satisfaction with clinic/ hospital care. Responses scales are 5-point scales. SAPS scores were interpreted as follows: 0 -10 = very dissatisfied, 11 - 18 = dissatisfied, 19 - 26 = satisfied, and 27 - 28 = very satisfied [17].

### Interventional pain procedure

We asked the patients to stop any medical treatment for the lumbar disc related radicular pain one week before the intervention. The selected patients were brought to the preparation room where reassurance was done. Intravenous midazolam 0.2 mg/kg was given to them, then they were placed in the prone position on fluoroscopy table and draped in a sterile manner. They were connected to a monitor (SPO2, NIBLP, and ECG) and given supplemental oxygen through a nasal cannula (3 L/min) to maintain an oxygen saturation. A 22-gauge, 3.5-inch spinal needle was used in the injection procedure. With each insertion of the spinal needle, 1 ml with 20 mg of local anesthetic lidocaine 2% was injected intradermally. Upon final needle-tip position, antero-posterior (AP) and lateral views of the fluoroscopic imaging were obtained to confirm needle positioning then the contrast was injected. AP and lateral views of the contrast spread were taken during the procedure to confirm appropriate spread into posterior epidural space.

The patients were randomly assigned into one of three groups:

1. Transforaminal injection of Mg sulphate:

In this group, the patients received transforaminal epidural injection of steroids and Mg sulphate with local anesthetic (7 mg Betamethasone, and 100 mg of magnesium sulphate diluted to 2 milliliters total volume with preservative-free normal saline, preceded by a test dose of 1 milliliter 2% lidocaine).

2. Transforaminal injection of Ozone:

In this group, the patients received transforaminal epidural injection of steroids (7 mg Betamethasone, preceded by a test dose of 1 milliliter 2% lidocaine) with the addition of an O2-O3 mixture, with an ozone concentration of 25 microgram /mL. We injected 3 mL of O2-O3 at transforaminal level.

3. Transforaminal injection of steroids alone:

In this group, the patients received transforaminal epidural injection of steroids with local anesthetic (7 mg Betamethasone preceded by a test dose of 1 milliliter 2% lidocaine).

After the intervention, the patients were not prescribed any medications (apart from NSAIDs for the first 3 days after injection) or specific physiotherapy regimens because they were already not responsive to them before the intervention.

### Laboratory assessment

Oxidative stress biomarkers (superoxide dismutase (SOD) and Glutathione (GSH)) were measured for all included patients in the three groups before and 2 weeks after the interventional procedure. For this purpose, the two-site sandwich ELISA method were employed. GSH and SOD levels were determined by reading the color changes compared to the standard curve in photometry. Samples were withdrawn from patients and the collected whole blood was refrigerated at 4 c for the night, then centrifuged it for 10 min at 1000-3000 rpm. The supernatant was taken and frozen at -80 c (for 1-3 months ) for storage.

Both markers SOD and GSH were measured by using double sandwich ELISA technique. The pre coated antibodies were human SOD and GSH monoclonal antibodies and the detecting antibody was biotin labeled polyclonal antibody. We added samples and biotin labeled antibody in ELISA wells then washed with TBS or PBS. We added Avidin peroxidase conjugate to the wells. We used TMB substrate for coloring after reactant thoroughly washed out by PBS or TBS. In presence of peroxidase activity TMB substrate changed into blue color. Finally, we added stop solution which changed blue color into yellow color. We measured the optical density (OD) by using ELIZA reader. The depth of the color and the testing factors in samples are positively correlated with their concentration. The standard curve was used to detect the amount of the biomarkers by plotting the average O. D for each standard against the concentration and drew a best fit curve using graph paper or statistical software analysis.

### Statistical analysis

Because our study was the first study to compare the therapeutic effect of transforaminal injection of Magnesium sulphate versus Ozone on the oxidative stress biomarkers in patients with lumbar disc prolapse, we calculated the sample size based on the results of a pilot study we performed before starting our study. The sample size calculation was done using G*Power version 3.1.9.7 Software. The probability of type I error (α) was 5%, effect size = 0.529, df =88, critical t= 1.66, noncentrality parameter λ= 2.51, A total sample size of 45 patients in each group was required to achieve a statistical power (1–β) 80%.

IBM SPSS (Statistical Package of Social Science) Version 25 was used to analyze the data. Categorical variables such as sex and the degree of the most prolapsed disc, were expressed as numbers and percentages. Quantitative variables such as age, duration of pain, the number of prolapsed discs, NRS, ODS, FRI, SAPS, SOD and GSH were expressed as mean and standard deviation. Chi-squared test was used for comparison between Mg sulphate, Ozone, and control groups in categorical variables, whereas One-way ANOVA was used for comparison between Mg sulphate, Ozone, and control groups in quantitative variables. Paired sample t- test was used for comparison between quantitative variables before and after the interventional pain procedure. Mixed ANOVA test was used for comparing quantitative variables before and after the interventional pain procedure in Mg sulphate, Ozone, and control groups. P-value ≤0.05 was considered statistically significant. All tests were two-tailed.

## Results

This prospective randomized controlled trial was carried out on 135 patients diagnosed as having symptomatic lumbar disc prolapse. Forty-five patients received transforaminal injection of Magnesium sulphate with steroids, 45 patients received transforaminal injection of Ozone with steroids, and 45 patients received transforaminal injection of steroids alone. There were no statistically significant differences between the three groups in either age or sex (*P*-value= 0.258, 0.392 respectively) (Table 1).

**Table 1:** Demographics, clinical, imaging and laboratory characteristics of the included patients and controls

		Mg sulphate group (*n*=45)	Ozone group (*n*=45)	Control group (*n*=45)	*P*-value
Age [Mean (SD)]		55.13 (13.22)	58.93 (13.28)	54.91 (12.37)	0.258
Sex	Males [n (%)]	27 (60%)	28 (62.2%)	22 (48.9%)	0.392
	Females [n (%)]	18 (40%)	17 (37.8%)	23 (51.1%)	
Duration of pain in months [Mean (SD)]		21.56 (14.96)	23.56 (15.56)	21.82 (14.39)	0.79
Number of prolapsed discs [Mean (SD)]		2.71 (0.99)	2.76 (0.98)	2.78 (0.95)	0.947
Degree of the most prolapsed disc	Bulge [n (%)]	21 (46.7%)	17 (37.8%)	16 (35.6%)	0.857
	Protrusion [n (%)]	16 (35.6%)	18 (40.0%)	19 (42.2%)	
	Herniation [n (%)]	8 (17.8%)	10 (22.2%)	10 (22.2%)	
NRS before intervention [Mean (SD)]		8.44 (1.24)	8.58 (1.29)	8.511 (1.12)	0.874
ODI before intervention [Mean (SD)]		62.13 (10.16)	59.96 (10.18)	62.84 (9.63)	0.363
FRI before intervention [Mean (SD)]		68.97 (17.2)	69.24 (18.02)	69.04 (16.98)	0.997
SAPS [Mean (SD)]		24.1556 (2.75)	23.33 (2.98)	21.67 (2.056)	>0.001*
SOD serum level before intervention [Mean (SD)]		12.4 (2.78)	12.12 (3.07)	12.75 (2.8)	0.593
GSH serum level before intervention [Mean (SD)]		24.46 (6.76)	25.42 (6.62)	24.61 (6.55)	0.763

*FRI* Functional rating index, *GSH Glutathione*, *NRS* Numeric rating scale, *ODI* Oswestry back disability, *SAPS* Short assessment of patient satisfaction, *SOD* Superoxide dismutase

*P-value ≤ 0.05 is considered significant

The clinical, imaging and laboratory characteristics of the patients in the three groups were demonstrated in Table 1. There was no statistically significant difference between the three groups in either the duration of pain, the number of prolapsed discs, the degree of the most prolapsed disc, NRS, ODI, FRI, SOD or GSH serum level before intervention. Both Magnesium sulphate and Ozone groups showed significantly higher SAPS scores in comparison to control group (*P*-value >0.001) (Table 1).

There was a statistically significant improvement in the scores of NRS, ODI and FRI, 2 weeks after intervention in the three groups, but at 1-month and 3-months after intervention, the significant improvement was in Mg sulphate and Ozone groups only. At 6-months follow up, Mg sulphate group only showed a significant improvement in the scores of NRS, ODI and FRI (Table 2).

**Table 2:** NRS, ODI, and FRI before and after Mg sulphate, Ozone and steroids injection

		Mg sulphate group (*n*=45) Mean (SD)	Ozone group (*n*=45) Mean (SD)	Control group (*n*=45) Mean (SD)
NRS	Before intervention	8.44 (1.24)	8.58 (1.29)	8.511 (1.12)
	After 2 weeks	5 (2.25)	4.98 (2.47)	8.02 (1.25)
	*P*- value	<0.001*	<0.001*	<0.001*
	*P*- value between groups	<0.001*		
	Before intervention	8.44 (1.24)	8.58 (1.29)	8.511 (1.12)
	After 1 months	4.29 (2.23)	4.13 (2.85)	8.38 (1.23)
	*P*- value	<0.001*	<0.001*	0.135
	*P*- value between groups	<0.001*		
	Before intervention	8.44 (1.24)	8.58 (1.29)	8.51 (1.12)
	After 3 months	3.24 (2.71)	3.62 (3.24)	8.4 (1.18)
	*P*- value	<0.001*	<0.001*	0.168
	*P*- value between groups	<0.001*		
	Before intervention	8.44 (1.24)	8.58 (1.29)	8.511 (1.12)
	After 6 months	4.09 (2.81)	8.4 (1.27)	8.49 (1.18)
	*P*- value	<0.001*	0.088	0.66
	*P*- value between groups	<0.001*		
ODI	Before intervention	62.13 (10.16)	59.96 (10.18)	62.84 (9.63)
	After 2 weeks	36.8 (10.94)	38.36 (17.16)	61.07 (10.4)
	*P*- value	<0.001*	<0.001*	0.005*
	*P*- value between groups	<0.001*		
	Before intervention	62.13 (10.16)	59.96 (10.18)	62.84 (9.63)
	After 1 months	23.69 (8.25)	30.58 (15.51)	61.93 (10.23)
	*P*- value	<0.001*	<0.001*	0.07
	*P*- value between groups	<0.001*		
	Before intervention	62.13 (10.16)	59.96 (10.18)	62.84 (9.63)
	After 3 months	23.6 (7.36)	23.73 (11.28)	61.69 (10.2)
	*P*- value	<0.001*	<0.001*	0.089
	*P*- value between groups	<0.001*		
	Before intervention	62.13 (10.16)	59.96 (10.18)	62.84 (9.63)
	After 6 months	25.02 (10.07)	60.11 (10.39)	61.76 (9.86)
	*P*- value	<0.001*	0.425	0.071
	*P*- value between groups	<0.001*		
FRI	Before intervention	68.97 (17.2)	69.24 (18.02)	69.04 (16.98)
	After 2 weeks	49.64 (17.28)	46.78 (19.14)	66.62 (17.33)
	*P*- value	<0.001*	<0.001*	0.001*
	*P*- value between groups	0.01*		
	Before intervention	68.97 (17.2)	69.24 (18.02)	69.04 (16.98)
	After 1 months	39.6 (15.46)	40.6 (18.19)	68.09 (17.19)
	*P*- value	<0.001*	<0.001*	0.189
	*P*- value between groups	<0.001*		
	Before intervention	68.97 (17.2)	69.24 (18.02)	69.04 (16.98)
	After 3 months	32.42 (15.57)	33.73 (17.01)	68.73 (17.41)
	*P*- value	<0.001*	<0.001*	0.654
	*P*- value between groups	<0.001*		
	Before intervention	68.97 (17.2)	69.24 (18.02)	69.04 (16.98)
	After 6 months	25.0889 (13.27)	68.87 (18.04)	68.98 (17.38)
	*P*- value	<0.001*	0.102	0.926
	*P*- value between groups	<0.001*		

*FRI* Functional rating index, *NRS* Numeric rating scale, *ODI* Oswestry back disability

*P-value ≤ 0.05 is considered significant

The number of patients who had ≥ 30% improvement of NRS and at least 15 points reduction in ODI score was significantly higher in both Mg sulphate and Ozone groups in comparison to the control group, at 2 weeks, 1- and 3-months following injection (*P*-value <0.001 in all comparisons). Whereas, after 6 months, this number was significantly higher in Mg sulphate group only in comparison to both Ozone and control groups (*P*-value <0.001) (Table 3).

**Table 3:** Comparison between Mg sulphate, Ozone and control groups regarding the improvement in NRS and ODI after the interventional pain procedure

		Mg sulphate group (*n*=45)	Ozone group (*n*=45)	Control group (*n*=45)	*P*-value
After 2 weeks					
NRS	Improved ≥ 30% [n (%)]	29 (64.4%)	29 (64.4%)	0	<0.001*
	Improved < 30% [n (%)]	16 (35.6%)	16 (35.6%)	45 (100%)	
ODI	Improved ≥ 15 [n (%)]	35 (77.8%)	29 (64.4%)	0	<0.001*
	Improved < 15 [n (%)]	10 (22.2%)	16 (35.6%)	45 (100%)	
After 1 month					
NRS	Improved ≥ 30% [n (%)]	35 (77.8%)	34 (75.6%)	0	<0.001*
	Improved < 30% [n (%)]	10 (22.2%)	11 (24.4%)	45 (100%)	
ODI	Improved ≥ 15 [n (%)]	41 (91.1%)	41 (91.1%)	0	<0.001*
	Improved < 15 [n (%)]	4 (8.9%)	4 (8.9%)	45 (100%)	
After 3 month					
NRS	Improved ≥ 30% [n (%)]	36 (80%)	33 (73.3%)	0	<0.001*
	Improved < 30% [n (%)]	9 (20%)	12 (26.7%)	45 (100%)	
ODI	Improved ≥ 15 [n (%)]	45 (100%)	44 (97.8%)	1 (2.2%)	<0.001*
	Improved < 15 [n (%)]	0	1 (2.2%)	44 (97.8%)	
After 6 month					
NRS	Improved ≥ 30% [n (%)]	31 (68.9%)	0	0	<0.001*
	Improved < 30% [n (%)]	14 (31.1%)	45 (100%)	45 (100%)	
ODI	Improved ≥ 15 [n (%)]	43 (95.6%)	0	0	<0.001*
	Improved < 15 [n (%)]	2 (4.4%)	45 (100%)	45 (100%)	

*NRS* Numeric rating scale, *ODI* Oswestry back disability

*P-value ≤ 0.05 is considered significant

There was a statistically significant increase in SOD and GSH serum levels, 2-weeks after intervention in both Magnesium sulphate (*P*-value = 0.002, 0.005 respectively) and ozone groups (*P*-value < 0.001, < 0.001), but there was no statistically significant change in SOD and GSH serum levels in control group (*P*-value = 0.059, 0.494 respectively) (Table 4).

**Table 4:** SOD and GSH serum level before and after Mg sulphate, Ozone and steroids injection

		Mg sulphate group (*n*=45) Mean (SD)	Ozone group (*n*=45) Mean (SD)	Control group (*n*=45) Mean (SD)
SOD serum level	Before intervention	12.4 (2.78)	12.12 (3.07)	12.75 (2.8)
	After 2 weeks	13.04 (2.93)	15.16 (3.44)	12.32 (2.63)
	*P*- value	0.005*	<0.001*	0.059
	*P*- value between groups	0.153		
GSH serum level	Before intervention	24.46 (6.76)	25.42 (6.62)	24.61 (6.55)
	After 2 weeks	26.1 (8.08)	30.99 (7.62)	24.42 (6.33)
	*P*- value	0.002*	<0.001*	0.494
	*P*- value between groups	0.031*		

*GSH*: *Glutathione*, *SOD* Superoxide dismutase

*P-value ≤ 0.05 is considered significant

## Discussion

The current study aimed to investigate the therapeutic effect of transforaminal injection of Mg sulphate versus Ozone on pain intensity, functional disability, and the oxidative stress biomarkers; SOD and GSH in patients with symptomatic lumbar disc prolapse.

Our results revealed that transforaminal injection of Mg sulphate caused a significant improvement in the scores of NRS, ODI and FRI, at 2 weeks, 1-month, 3-, and 6-months follow-up.

In accordance with our findings, a long-term study of magnesium supplementation for patients with chronic refractory low back pain showed a significant reduction in pain intensity and improvement in lumbar spine mobility at 6 months follow up [18].

Also, Demiroglu et al. found that using magnesium sulphate regionally at surgical postoperative laminectomy can decrease postoperative lumbar pain even more than systemically administered magnesium [19].

Magnesium has an indirect anti-nociceptive function as it blocks NMDA receptors that found centrally and peripherally transferring pain signal, thus it prevents central sensitization and decrease pain threshold caused by peripheral tissue injury [20]. Magnesium is also known to antagonize the expression of some inflammatory mediators (serotonin, histamine, and some cytokines) [21].

The significant long-term improvement (up to 6 months) in pain and functional disability in patients who received Mg sulphate in our study can be attributed to the neuroprotective and anti-inflammatory effects of Mg sulphate. The neuro-protective effect of magnesium sulfate has been one of the most challenging aspects of this drug. Magnesium was reported to plays a fundamental role in neuroplasticity. It is directly involved in the maintenance of neurological integrity and neuroprotection through reduction in glutamate release and inhibiting calcium entry into the cell via a noncompetitive blockade of NMDA receptor [22–24]. It also prevents activation of catabolic enzymes (e.g. phospholipases, proteases, and endonucleases), production of free radical and the secondary cascade of neuronal injury that leads to apoptosis [25, 26]. Magnesium sulfate may confer neuroprotection through downregulation of the inflammatory cascade. It reduces the production of the pro-inflammatory cytokines; interleukin-6 and tumor necrosis factor-α, substance P in addition to suppressing the Nuclear Factor kB (NF-kB) activation [27, 28].

In contrast to these findings, another study revealed that infusion of Mg sulfate in patients undergoing laminectomy didn’t produce a clinically significant long-term improvement in postoperative pain severity. It showed only improvement for very short period (24 hours) [29].

Our results revealed that transforaminal injection of ozone caused a significant improvement in the scores of NRS, ODI and FRI, at 2 weeks, 1-, and 3-months follow-up.

Ozone therapy has been used in multiple studies as an additional treatment option in patients with lumbar disc prolapse. The proximal injection of the O2-O3 gas mixture to the root ganglion can normalize the levels of prostaglandins and cytokines, decrease reactive oxidant species levels, increase SOD enzyme activity, and improve the circulation around the root ganglion [30].

In a randomized trial carried by Niu et al.(2018), the therapeutic effect of different concentrations of medical ozone on pain severity and disc retraction was investigated in patients with trauma-induced lumbar disc herniation. There was a significant disc retraction and decrease in inflammatory markers and pain severity at all groups using different ozone concentrations [31].

Hosseini et al.(2019) performed oxygen-ozone chemonucleolysis on a large number of patients with lumbar disc herniation and registered a high success rate at all types of disc herniation and failed back surgery [32]. Also several meta-analyses were published indicating the efficacy of intradiscal ozone injection in patients with disc prolapse [33, 34].

Gallucci et al. (2007) mentioned in their study the superiority of ozone injection over steroid injection at 6-months follow-up [35]. In contrast to these findings, Ryska et al. didn’t reveal significant differences in the therapeutic effectiveness between transforaminal ozone and steroid injection at three- and six-months follow-up [36].

Our study revealed a significant increase in SOD and GSH serum levels, 2 weeks after intervention in both Mg sulphate and Ozone groups, but there was no statistically significant change in SOD and GSH serum levels in control group.

It is well known that the development and progression of disc herniation and degeneration are closely associated with oxidative stress and reactive oxygen species (ROS). To prevent this damage, a lot of biological defense mechanisms occurs including increased antioxidant enzymes like SOD, and GSH synthesis. It was found that the activity of SOD is decreased in advanced rat lumbar inter-vertebral disc degeneration [37–39].

The beneficial effect of Mg sulphate on oxidative stress has been proven by several studies [40, 41]. Mg reported to be present in more than 300 enzymes. It is well known to alter the neuronal sensitivity to an oxidative insult [42]. It has been demonstrated that treatment with MgSO_4_ attenuates oxidative damage and ROS generation [43].

Ozone therapy has been also identified as a promising therapeutic intervention that have the ability to attenuate oxidative damage. Ozone administration was reported to stimulate the antioxidant system by upregulating some antioxidant enzymes such as SOD, GSH-peroxidases, transferases, and reductases [44].

Regarding patients’ satisfaction our results revealed a significant superiority of both transforaminal injection of Mg sulphate and Ozone over steroids at 6-months follow-up.

The strength of our study is that it is the first study to investigate the therapeutic effect of transforaminal injection of Magnesium sulphate versus Ozone on the clinical outcome and the oxidative stress biomarkers; SOD and GSH in patients with symptomatic lumbar disc prolapse

The main limitation of our study was that we didn’t measure the serum level of SOD and GSH at 1-, 3- and 6-months follow-up. We also didn’t do electrophysiological assessment for the included patients before and after injection. Another limitation was that we didn’t investigate the effect of transforaminal injection of either Mg sulphate or Ozone on the markers of neurodegeneration and neuroplasticity.

## Conclusion

Each of transforaminal injection of Mg sulphate and ozone results in a significant improvement in pain intensity and functional disability in patients with symptomatic lumbar disc prolapse at 2 weeks, 1-, and 3-months follow-up. Transforaminal injection of Mg sulphate causes significant long-term improvement (up to 6 months) in pain intensity and functional disability. The serum levels of the anti-oxidants; SOD and GSH were significantly increased at 2 weeks following both transforaminal injection of Mg sulphate and ozone.

## Data Availability

Authors report that the datasets used and/or analyzed during the current study are available from the corresponding author on reasonable request.
